# An Endophytic Bacterial Strain Isolated from *Eucommia ulmoides* Inhibits Southern Corn Leaf Blight

**DOI:** 10.3389/fmicb.2017.00903

**Published:** 2017-05-18

**Authors:** Ting Ding, Bo Su, Xiaojie Chen, Shanshan Xie, Shuangyue Gu, Qi Wang, Dayue Huang, Haiyang Jiang

**Affiliations:** ^1^School of Plant Protection, Anhui Agricultural UniversityHefei, China; ^2^Key Laboratory of Crop Biology of Anhui Province, Anhui Agricultural UniversityHefei, China

**Keywords:** endophytic bacteria, southern corn leaf blight, lipopeptides, colonization, induced systemic resistance

## Abstract

*Bacillus subtilis* DZSY21 isolated from the leaves of *Eucommia ulmoides* oliv. was labeled by antibiotic marker and found to effectively colonize the leaves of maize plant. Agar diffusion assays and biocontrol effect experiments showed that strain DZSY21 and its lipopeptides had antagonistic activity against *Bipolaris maydis*, as well as high biocontrol effects on southern corn leaf blight caused by *B. maydis*. Using MALDI-TOF-MS analysis, we detected the presence of antimicrobial surfactin A, surfactin B, and fengycin in the strain DZSY21. Signaling pathways mediated by DZSY21 were analyzed by testing the expression of key plant genes involved in regulation of salicylic acid (SA) or JA/ET pathways, the defense-related genes *PR1* and *LOX* were concurrently expressed in the leaves of DZSY21-treated plants; this corresponded to slight increase in the expression level of *PDF1.2* and decreases in *ERF* gene transcription levels. The results indicated an induced systemic response that is dependent on the SA and jasmonic acid (JA) pathways. Thus, we hypothesized that the strain DZSY21 inhibits *B. maydis* by producing antifungal lipopeptides and activating an induced systemic response through SA- and JA-dependent signaling pathways. This work describes a mechanism behind reduced disease severity in plants inoculated with the endophytic bacteria DZSY21.

## Introduction

Epidemics of southern corn leaf blight in maize are caused by *Bipolaris maydis*; these infections are regarded as one of the most destructive foliar diseases due to their extensive damage to crop yield and quality (Wang et al., 2015). Traditionally, fungicides and resistant cultivars have been used to control this disease in the field. Some fungicides, such as Chlorothalonil, Carbendazim, and Thiophanate-methyl wettable powders are effective in preventing disease (Akgül and Mirik, 2008); however, the use of chemical fungicides pollutes the environment and catalyzes the development of fungicide-resistant strains (Bajwa et al., 2003). Therefore, there is an interest in technologies that would reduce dependency on chemical pesticides. Biological pest control, including the use of microorganisms to control plant diseases, offers an attractive alternative that would alleviate many of the negative impacts of chemicals.

Endophytic bacteria are micro-organisms that colonize healthy plant tissue without causing any apparent symptoms or diseases to the host (Arnold et al., 2003). These strains could exert several beneficial effects on host plants, including conferring resistance against different biotic and abiotic stresses (Kharwar et al., 2008), inducing resistance to plant pathogens, and producing beneficial bioactive substances. There is interest in the use of endophytic bacteria for the biological control of plant diseases (Nejad and Johnson, 2000; Verhagen et al., 2004; Compant et al., 2005). So far, a variety of endophytes had been reported to confer protection against bacterial and fungal pathogens (Lodewyckx et al., 2002; Sessitsch et al., 2004). However, not enough is known about the mechanisms by which endophytic bacteria confer benefits. Understanding the mechanisms of biocontrol is critical to improving the efficacy of and implementing the use of biocontrol agents. The object of this study was to understand the inhibitory mechanisms by endophytic bacteria confer protection against disease.

*Bacillus* species are among the most common endophytic bacteria (Bacon and Hinton, 2002; Choudhary and Johri, 2009), and there are many reports describing the ability of *Bacillus* spp. to suppress several important plant pathogens (Melo et al., 2009). In recent years, *Bacillus* spp. have been used as a biocontrol agent to protect crops against plant diseases, and provide an alternative to chemical fungicides (Augustine et al., 2010; Yang et al., 2012; Zhou et al., 2014). The main biocontrol mechanisms of *Bacillus* spp. are considered to be the production of antibiotics (direct) (Ongena et al., 2005; Ongena and Jacques, 2008), such as lipopeptides which aroused great attention for suppressing the growth of fungal pathogens and stimulating the innate immunity of plant system against various pathogens (Ongena et al., 2007; Romero et al., 2007; Raaijmakers et al., 2010), the competition for ecological niches (direct) (Compant et al., 2005), or the induction of systemic resistance (ISR) in host plants (indirect) (Van Loon and Bakker, 2006; Saravanakumar et al., 2007). However, the effectiveness of endophytes as biological control agents (BCAs) is dependent on efficient colonization of the plant environment. The extent of endophytic colonization in host plant organs and tissues reflects the ability of bacteria to selectively adapt and compete in those specific ecological niches.

*Eucommia ulmoides* oliv. was a medicinal plant in southern China, which was known for hosting several metabolites having medicinal property (Matsuda et al., 2006; Zhang et al., 2014), and many studies on it had been carried out in terms of some products of secondary metabolism (Matsuda et al., 2006; Zhang et al., 2014). However, reports on the antagonistic endophytic bacteria isolated from the *E. ulmoides* oliv. and their potential to promote plant disease resistance were relatively few. In this study, the *Bacillus subtilis* DZSY21 isolated from the leaves of *E. ulmoides* oliv. exerts a strong antifungal effect on *B. maydis*. On that basis, we investigated the inhibition mechanisms of the biocontrol strain DZSY21, including direct antagonism and induced systematic resistance, as well as the ability of DZSY21 to colonize maize leaves. This work provides a theoretical basis for the use of DZSY21 as a replacement for pesticides and supplements.

## Materials and Methods

### Endophytic Bacterial Strain and Phytopathogen

The leaves of *E. ulmoides* oliv., which were collected at Anhui Agricultural University (31°86′ N and 117°25′ E) in the Anhui Province of China, were firstly washed in running water, and dipped in 70% ethanol for 1 min and then treated with 1% sodium hypochlorite for 10 min. The samples were then washed several times with sterilized distilled water, the final wash was spread plated onto nutrient agar plate (g/L; peptone 5, beef extract 2, yeast extract 3, sodium chloride 5 and agar 18, pH 7.0) and cultivated at 28°C for 3 days as a sterility check. Samples were discarded if the growth was detected in the sterility check samples after 3 days (Tiwari et al., 2010; Sun et al., 2013).

For the isolation of endophytic bacterium DZSY21, 1 g of leaf tissues were fully grinded to homogenate in 9 ml sterilized distilled water in a mortar, 100 μL of the extract was taken and serial diluted up to 10^-3^ dilution. Then, 100 μL was plated onto nutrient agar plate with three replications. The plates were incubated at 28–30°C for 48–72 h. Each bacterium, as evident from their colony morphology was transferred to fresh nutrient agar medium plates to establish pure culture of endophytic bacterium. Lastly, endophytic bacterium DZSY21 was obtained and identified as *Bacillus subtilis*, the sequence data has been submitted to GenBank (accession No. KP777560). The strain was grown at 30°C for 24 h in beef-protein medium (beef extract 3 g/L, peptone 10 g/L, NaCl 5 g/L, pH 7.0–7.2). The cell density was adjusted to approximately 10^8^ CFU/mL in sterile distilled water for use.

The target phytopathogenic fungal strain, *B. maydis*, was obtained from the School of Plant Protection at Anhui Agricultural University. The strain was maintained on potato dextrose agar medium (potato 200 g/L, dextrose 20 g/L, agar 15 g/L). Conidia of *B. maydis* was induced on niblet culture (niblet 80 g, H_2_O 10 mL) incubated at 28°C for 10 days under a 12-h of light/dark cycle. The cultures were washed in sterile water and the mycelia were filtered through four layers of gauze to obtain conidia suspension. The concentration of the conidia suspension was adjusted to approximately 10^5^ CFU/mL using a hemocytometer.

### Extraction of Lipopeptides

DZSY21 was grown in Landy medium (Glucose 20 g/L, L-glutamic acid 5 g/L, MgSO_4_ 0.5 g/L, KCl 0.5 g/L, KH_2_PO_4_ 1 g/L, FeSO_4_⋅6H_2_O 0.15 mg/L, MnSO_4_ 5.0 mg/L, CuSO_4_⋅5H_2_O 0.16 mg/L, pH 7.0) at 30°C and 180 rpm in a shaker for 38 h. After centrifugation (5000 rpm, 15 min), and cell-free supernatants (adjust to pH 2.0 by with 6 M HCl) were incubated overnight at 4°C. The acid precipitate was collected by centrifugation (8000 rpm for 10 min) and extracted twice with methanol. The methanol extracts of acid precipitate were lipopeptides and the pH was adjusted to 7.0 with NaOH (2 M). The lipopeptides were concentrated using a vacuum freeze drier, and the dried material was dissolved in a suitable volume of methanol for further analysis.

### Antifungal Assays of the Strain DZSY21 and its Lipopeptides

The inhibitory effect of endophytic bacterium DZSY21 on the growth of *B. maydis* was evaluated by the plate dual-culture method (Kunova et al., 2016). Firstly, a 5-mm mycelium disk cut from a 5-day-old culture of *B. maydis* was placed on the center of Petri dish, and cultivated for 3 days in advance, then strain DZSY21 from 2-day-old culture was streaked across approximately 2.5 cm away from the disk in the center of the plates. Water was used as negative control. The plates were continued to incubate at 28°C for 4 days. The inhibitory activity of treatment was carried out using the following formula, where DC = radius of control, and DT = radius of fungal colony with treatment. The experiments were repeated in triplicate and the data presented here were the averages of three experiments.

$$\text{Growth inhibition}(\%)\;=\;\frac{DC-DT}{DC}\;\times \;100\%$$

The antifungal activity of lipopeptides was evaluated by disk diffusion assay (Bauer et al., 1966). Firstly, the disk of *B. maydis* was placed on the center of Petri dish and incubated for 3 days in advance, then filter paper disks (5 mm) were switched with 300 μg of lipopeptides and placed in approximately 2.5 cm away from the disk, a methanol switched disk was used as the control. The plates were continued to incubate at 28°C for 4 days. Antifungal activity was determined by observing the inhibition of fungal growth around the disk. Then the fungal mycelia treated with the DZSY21 and its lipopeptides was examined under a microscope.

### Colonization Studies on Maize Leaves

To study the ability of DZSY21 to colonize maize leaves, the *Bacillus subtilis* DZSY21 was labeled by antibiotic marker (Chen et al., 1995; Nautiyal et al., 2002; Bennett et al., 2003; Wang et al., 2010a). Firstly, the sensitive concentrations of the strain DZSY21 to antibiotics were analyzed, and sensitive concentrations of the strain DZSY21 to kanamycin and chloramphenicol were 7.5 and 1 μg/mL, respectively. On that basis, the DZSY21^Kan^ was obtained by transferring colonies to LB medium agar plates containing increasing concentrations of kanamycin (Serva; 5, 10, 15, 20, 25, 50, 100, 150, and 200 μg/mL), then the DZSY21^Kan^ was transferred to LB medium agar plates containing increasing concentrations of chloramphenicol (0.5, 1, 5, 10, and 15 μg/mL) and fixed concentration of kanamycin (200 μg/mL), lastly, the double-resistance strain DZSY21^Kan,chl^ was obtained. The stability and antagonistic effect of the DZSY21^Kan,chl^ were tested by 20 sub-cultures on LB agar with kanamycin (200 μg/mL) and chloramphenicol (15 μg/mL), incubating for 48 h at 30°C, the stability of the DZSY21^Kan,chl^ was determined by comparing the number of CFUs after the last subculture, the antagonistic effect was evaluated through the plate confrontation method. And the DZSY21^Kan,chl^ was characterized by amplification and sequencing of a partial sequence of the 16S rDNA gene. The DZSY21^Kan,chl^ were stored at 4°C on LB with kanamycin (200 μg/mL) and chloramphenicol (15 μg/mL).

To determine whether the strain could colonize plant leaves, maize leaves inoculated with the suspensions (1 × 10^8^ CFU/mL) of the double-resistance strain DZSY21^Kan,chl^ (50 mL per plant) and incubated for 24 h, maize leaves treated with sterile water were used as a control (50 mL per plant). The samples were collected at 24 h post-inoculation, they were treated and observed by transmission electron microscopy at biotechnology center of Anhui Agricultural University.

To analyze the population density of the strain in maize leaves, at the pumping stage of maize plant, suspensions of the double-resistance strain DZSY21^Kan,chl^ were adjusted to 10^8^ CFU/mL in sterile distilled water, and 50 mL/plant was applied to the maize leaves in each plant. Maize leaves treated with sterile water were used as a control (50 mL per plant). Leaves were harvested at 1, 3, 5, 7, 10, 15, 20, 25, and 30 days after inoculation, and the population of the DZSY21^Kan,chl^ inside the mesophyll tissue was estimated. The maize leaves were weighed, cut into small pieces, surface sterilized with 0.1% corrosive sublimate for 1 min, treated with 1% sodium hypochlorite for 10 min, and washed several times with sterilized distilled water. The sterilized plant material was trimmed, ground, and diluted with phosphate buffer saline (PBS) (sodium chloride 8 g/L, potassium chloride 0.2 g/L, disodium hydrogen phosphate 1.44 g/L, and potassium dihydrogen phosphate 0.24 g/L; pH 7.4) up to a dilution factor of 10^-3^. 100 μL of each dilution was plated on LB medium containing kanamycin (200 μg/mL) and chloramphenicol (15 μg/mL). The plates were counted after 48 h incubation at 30°C. This experiment was conducted in duplicate.

### Biocontrol Assays

Plot experiments were carried out in the teaching practice base of Anhui Agricultural University in July 2015. Maize seedlings (Chang 7-2, F1) were grown in the soil with planting depth of 10 cm. The soil was tilled twice before pre-sterilized (3% sodium hypochlorite solution) maize seeds were planted. At the pumping stage, the maize plants were divided into four plots, and 40 plants *were* present in one plot. The efficacy of DZSY21 in suppressing southern corn leaf blight was evaluated by applying lipopeptides (1 mg/mL) and DZSY21 suspensions (1 × 10^8^ CFU/mL) by spraying the leaves of the maize plants (50 mL per plant). Sterile water and 50% carbendazim wettable powder 600 times liquid were used as the negative and positive controls, respectively. The conidial suspension of *B. maydis* (1 × 10^5^ CFU/mL) was applied to the leaves via the same mechanism 24 h after application of the treatment; the plants were then moistened with a humidifier for 12 h. Each treatment was replicated 40 times and the experiment was repeated three times. The disease severity was recorded 4, 6, 8, 10, and 15 days after challenge with the pathogen according to a rating scale of 1–9 scales with 1 being the most resistant and 9 being dead (Balint-Kurti et al., 2007), and the Disease index and disease reduction were calculated according to the formulas below.

$$\begin{array}{c} \text{Disease index}\;=\;\frac{\Sigma ({\text{d}}_{\text{i}}\times {\text{l}}_{\text{i}})}{\text{L}\times \text{N}}\;\times \;100\\ \text{Disease reduction}\;=\;({\text{I}}_{\text{0}}-{\text{I}}_{\text{i}})\;\times \;100\% \end{array}$$

where, d_I_ = represents for the grade of disease severity, l_I_ = the number of leaves at different grades of disease, L = the number of all investigated leaves, N = the highest grade of disease severity, I_0_ = the disease index of control, and I_I_ = the disease index of different treatment groups. As a control, sterile water was spread on leaves.

### Molecular Mass of DZSY21 Lipopeptides Determined by MALDI-TOF

Lipopeptides were analyzed for surfactin, iturin, and fengycin using MALDI-TOF-MS. A sample (1 mg/mL) was diluted 10x with 100% methanol, and data was acquired in positive reflector mode from 800 to 4000 m/z. The analysis was performed at biotechnology center of Anhui Agricultural University.

### Study of Plant Defense Response

The lower leaves (i.e., the first leaf to the fourth leaf) of axenic maize plants from nine different leaf periods were covered with DZSY21 (5 mL per leaf of 10^8^ CFU/mL) and cultivated in the greenhouse (30–35°C) under a 12-h-light and 12-h-dark interval. Control plants were covered with 5 mL (per leaf) of sterile water instead of the bacterial cell suspension. Then the bacterized and non- bacterized leaves from the upper part of the plants (i.e., the sixth leaf to the ninth leaf) were harvested at 12, 24, 36, 48, and 60 h for RNA extraction. Forty-five plants were present in each treatment, nine maize plants were harvested and divided into three replicates at different period, and each sample was mixtures of the upper leaves of the three maize plants.

Total RNA was extracted using the Trizol reagent (Invitrogen) according to the manufacturer’s instructions. The DNase-treated RNA was reverse-transcribed using M-MLV reverse transcriptase (Invitrogen). The primer pairs used for the qRT-PCR analysis were designed according to parameters established for the Primer3Plus program (Untergasser et al., 2007; Klosterman et al., 2011) (**Table 1**). Expression of pathogenesis-related protein 1 (*PR1*), defensin (*PDF1.2*), lipoxygenase (*LOX*), and ethylene response factor (*ERF*) were monitored by qRT-PCR in plants in response to DZSY21 treatment. The candidate reference genes actin was identified as the most stable gene and was used as an endogenous control in qRT-PCR analysis. qRT-PCR was performed on an ABI 7300 Real-Time System (Applied Biosystems). Each reaction contained 12.5 μL of 2× SYBR Green Master Mix reagent (Applied Biosystems), 400 nM of gene-specific primers, and 1.5 μL of diluted cDNA sample (final volume 20 μL). The thermocycle conditions were: 95°C for 10 min, followed by 40 cycles of 95°C for 15 s and 60 °C for 60 s. After the PCR was complete, a melting curve was generated to analyze the specificity for each gene by increasing the temperature from 60 to 95°C. Three replicates were performed for each gene. Results are based on the average of triplicates, and the standard deviation of the mean is shown.

**Table 1:** Primer sequences used for quantitative polymerase chain reactions.

Gene	GenBank accession number	Primer name	Sequence
PR-1	U82200.1	PR-1-FPR-1-R	5′-AACAATGGCACCGAGGCT-3′5′-GTAGTCCTGCGGCGAGTT-3′
PDF1.2	JF797205.1	PDF 1.2-FPDF 1.2-R	5′-CCTCGTCCTCATGCTCCTCC-3′5′-ATGAGCCCGATGCTGGTG-3′
LOX	NM_001111533.2	LOX-FLOX-R	5′-AGGAGTTTGGACGGGAGATT-3′ 5′-CCGTACTTGCTCGGGTCA-3′
ERF	NM_001155962.1	ERF-FERF-R	5′-TAAGAGGTCTGCGGCTAACA-3′5′-TCATCGTCCCAGTCCCAC-3′
Actin	DQ492681.1	Actin-FActin-R	5′-CGACTGCTGAGCGAGAA-3′5′-TGAAGGATGGCTGGAATA-3′

### Statistical Analysis

The data of all experiments was analyzed by analysis of variance (ANOVA). When ANOVA showed treatment effects (*P* < 0.05), the least significant difference test (LSD) and Duncan’s multiple range test (for maize) were applied to make comparisons among the means. The statistical package DPS ver. 9.50 was used for all analyses.

## Results

### Antifungal Activity of the Strain DZSY21 and Its Lipopeptides

Lipopeptides are cyclic, low molecular weight antimicrobial compounds, which are mainly composed of a 7–10 amino acid hydrophilic head linked with a hydrophobic fatty acid tail (Cai et al., 2013). *Bacillus* species have strong antimicrobial properties and are known to produce a structurally diverse group of antimicrobial lipopeptides, including surfactin, iturin, and fengycin families (Wang et al., 2010b; Cai et al., 2013).

**FIGURE 1:**
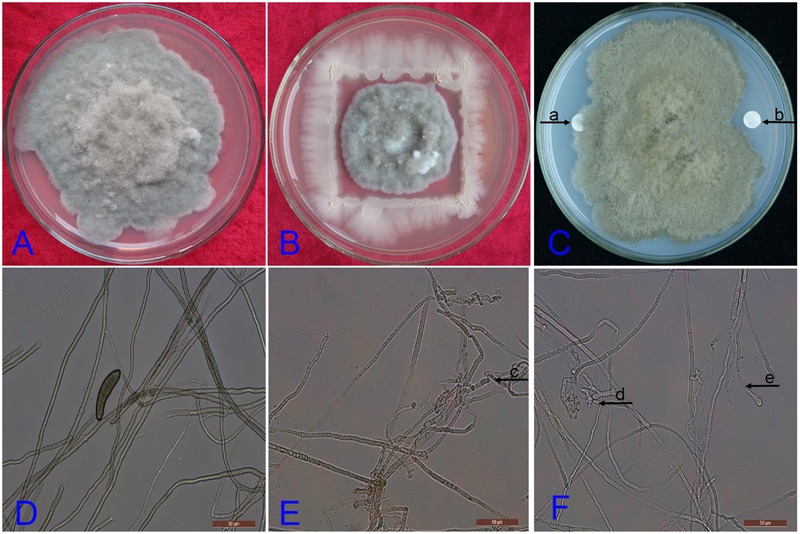
**Antifungal activity of *Bacillus subtilis* DZSY21 and its lipopeptides: (A)** the colony of *Bipolaris maydis* treated with water incubated for 7 days; **(B)** the colony of *B. maydis* treated with the strain DZSY21 incubated for 7 days; **(C)** filter paper disks (5 mm) were switched with 300 μg lipopeptides (**a**, filter paper disk switched with methanol; **b**, filter paper disk switched with lipopeptides); **(D)** microscopic images showing mycelium morphology of *B. maydis* treated with water; **(E)** microscopic images showing deformation in DZSY21 treated culture (**c** is the expansion and rupture of hyphae), and **(F)** microscopic images showing deformation in lipopeptides treated culture (**d** and **e** are the expansion and rupture of hyphae, respectively).

Screening of the endophytic strain DZSY21 for antifungal activity by the dual culture assay method showed antifungal activity by inhibition of fungal colony growth. After being cultured for 7 days, the diameter of the colony of the *B. maydis* treated with the strain DZSY21 was roughly 3 cm (**Figure 1B**), while the colony of *B. maydis* treated with water was basically full of Petri dish (**Figure 1A**). The strain DZSY21 exhibited antifungal activity against *B. maydis* with inhibition 61.70%.

The isolated endophytic strain DZSY21 was grown in Landy medium for 38 h in order to induce secretion of antifungal lipopeptides. Cell-free supernatant was collected by centrifugation (5000 rpm, 15 min) at 4°C, the acid precipitate was obtained from cell-free supernatant by adding concentrated HCl to reduce pH at 2 and incubated overnight at 4°C, the methanol extracts of acid precipitate were lipopeptides. The lipopeptides were assayed against the fungal pathogen *B. maydis* by disk diffusion assay. After being cultured for 7 days, the lipopeptides from DZSY21 exhibited antifungal activity against *B. maydis*, there was an obvious inhibition belt between the fungal colony and the disk with lipopeptides (**Figure 1C-b**), while the disk switched methanol was covered with hyphae of *B. maydis* (**Figure 1C-a**), the disk switched methanol had not antifungal activity against *B. maydis*. Microscopic examination of affected mycelia showed the DZSY21 could cause the mycelium inflation (**Figure 1E-c**), and some mycelia treated with lipopeptides were swelled, contorted (**Figure 1F-d**) and broken (**Figure 1F-e**). Meanwhile, the mycelium in the control plates (i.e., only treated with methanol) were smooth, vimineous, and evenly grown (**Figure 1D**).

### Ability of DZSY21 to Colonize Maize Leaves

**FIGURE 2:**
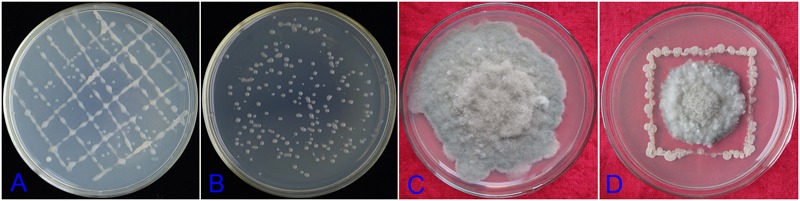
**Morphology and antifungal activity of the strain DZSY21^Kan,chl^: (A)** the morphology of the strain DZSY21; **(B)** the morphology of the strain DZSY21^Kan,chl^; **(C)** the colony of *B. maydis* treated with water incubated for 7 days; **(D)** the colony of *B. maydis* treated with strain DZSY21^Kan,chl^ incubated for 7 days.

To investigate colonization ability of the endophytic strain DZSY21 in maize leaves, DZSY21 was tagged with kanamycin and chloramphenicol. The double-resistance strain DZSY21^Kan,chl^ was selected on LB agar with kanamycin (200 μg/mL) and chloramphenicol (15 μg/mL). After approximately 20 generations of growth in the antibiotic medium with kanamycin (200 μg/mL) and chloramphenicol (15 μg/mL), the stability of the DZSY21^Kan,chl^ were evaluated by comparing the number of CFUs after the last subculture, and analyzing the characteristics of colony of DZSY21^Kan,chl^ and DZSY21. The results indicated the colonies of the DZSY21^Kan,chl^ (**Figure 2B**) and the strain DZSY21 (**Figure 2A**) were all smooth, moist and milky white, and the number of CFUs of the DZSY21^Kan,chl^ in different culture generation had no obvious difference. Additionally, the diameter of colony of *B. maydis* treated with the strain DZSY21^Kan,chl^ (**Figure 2D**) was similar to that of the DZSY21 (**Figure 1B**), while the colony of *B. maydis* treated with water was basically full of Petri dish(**Figure 2C**). The antifungal activity of DZSY21^Kan,chl^ was stable with 67.00% inhibition, compared to 61.85% for the wild-type strain DZSY21. And the DZSY21^Kan,chl^ was also identified as *Bacillus subtilis.* Therefore, the double-resistance strain DZSY21^Kan,chl^ was regarded as the mutant of DZSY21, the colonization ability of the DZSY21 in maize leaves was clarified through utilizing strain DZSY21^Kan,chl^.

**FIGURE 3:**
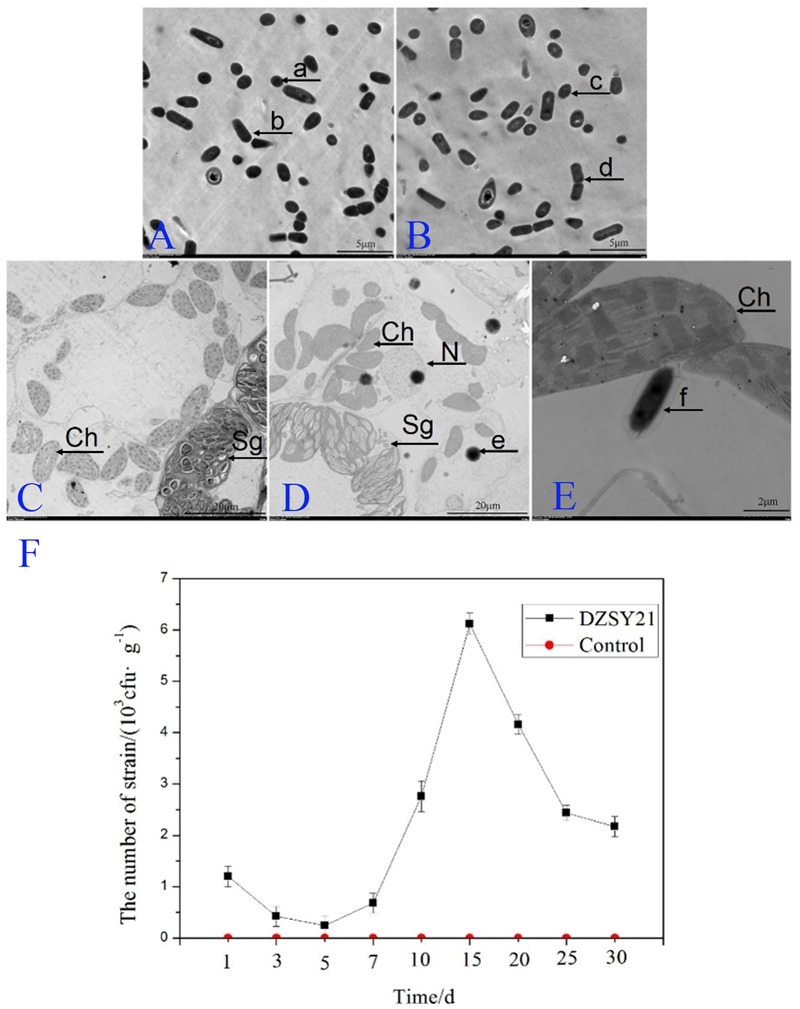
**Development of the strain DZSY21^Kan,chl^ in the maize leaves 24 h post-inoculation: (A)** the different shapes of the wild-type DZSY21 observed by transmission electron microscopy (**a**, the spherical DZSY21; **b**, the rod-shaped DZSY21); **(B)** the different shapes of DZSY21^Kan,chl^ observed by transmission electron microscopy(**c**, the spherical DZSY21^Kan,chl^; **d**, the rod-shaped DZSY21^Kan,chl^); **(C)** TEM photographs of maize leaf tissue treated with water; **(D,E)** TEM photographs of maize leaf tissue treated with the DZSY21^Kan,chl^ (Ch, chloroplast; N, Nuclear; Sg, Starch grain; **e** and **f**, the DZSY21^Kan,chl^), and; **(F)** population dynamics of the DZSY21 mutant on maize leaf tissue.

Understand the shape of the DZSY21^Kan,chl^ by transmission electron microscopy was in favor of estimating whether the strain could colonize plant leaves. The DZSY21^Kan,chl^ was observed by transmission electron microscopy and presented rhabditiform and globosity (**Figure 3B**), the shape of the strain DZSY21^Kan,chl^ was the same as that of the wild-type DZSY21 (**Figure 3A**). Then maize leaves were inoculated with the suspensions (1 × 10^8^ CFU/mL) of the double-resistance strain DZSY21^Kan,chl^ (50 mL per plant), 24 h after inoculation with the DZSY21^Kan,chl^, bacterial cells were found to be localized to the intercellular spaces of leaf tissues. Furthermore, colonization of the DZSY21^Kan,chl^ in maize leaves did not have any significant effects on the cell tissues of leaves, chloroplast and starch grains were normal, and the bacterial strain and maize leaves formed a harmonious endophytic relationship (**Figures 3D,E**). No bacteria were observed in the control plants (**Figure 3C**). Using the dilution plating method, the population density of the DZSY21^Kan,chl^ in maize leaves was detected, the population density reached 6.35 × 10^3^ CFU/g leaf tissue at 15 days post-inoculation, and remained above 2.17 × 10^3^ CFU/g leaf tissue until 30 days post-inoculation. No labeled strains could be isolated from the control plants during the course of the experiments (**Figure 3F**). Quantification studies revealed the maize leaves were successfully colonized by DZSY21.

### Biocontrol on Southern Corn Leaf Blight with DZSY21

**FIGURE 4:**
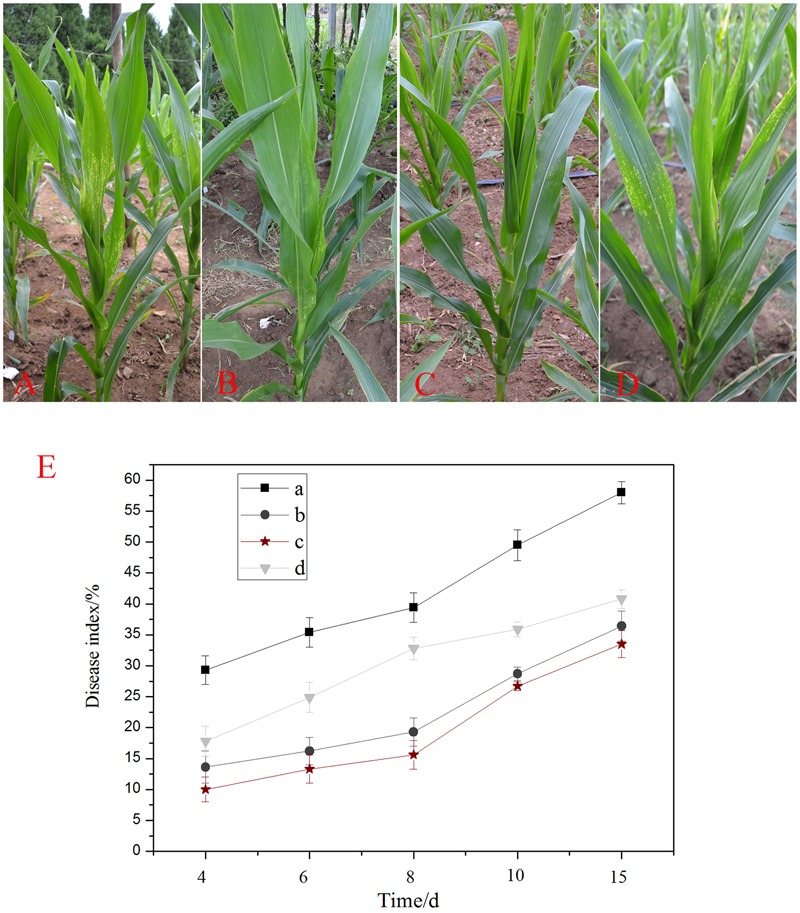
**Induction of the systemic resistance in maize plants by *Bacillus subtilis* DZSY21: (A)** plants drenched with a conidial suspension of *B. maydis*; **(B)** plants drenched with lipopeptides and then challenged with a conidial suspension of *B. maydis*; **(C)** plants drenched with suspensions of DZSY21 and then challenged with a conidial suspension of *B. maydis*; **(D)** plants drenched with carbendazim wettable powder and then challenged with a conidial suspension of *B. maydis*; **(E)** the graph of disease index of different groups, leaves incubated for 24 h with **(a)** water control, **(b)** lipopeptides, **(c)** suspension of endophytic strain DZSY21, and **(d)** 50% carbendazim wettable powder and then challenged with *B. maydis*. Disease index was calculated at different days after challenge with *B. maydis*. Data are expressed as the average of three replicates ± standard deviation.

To verify if *Bacillus subtilis* DZSY21 was able to induce resistance in maize, which might be an indirect factor promoting maize growth, we sprayed the lipopeptides (1 mg/mL) and DZSY21 suspensions (1 × 10^8^ CFU/mL) on the leaves of the maize plants (50 mL per plant), and subsequently challenged leaves with the pathogen *B. maydis*, from the fourth day after the challenge, the symptoms of southern corn leaf blight appeared in all groups, with the prolonging of time of growth, there were obvious differences in the symptoms of maize leaves in different groups. Compared with the negative control (**Figure 4A**), the plants inoculated with lipopeptides (**Figure 4B**), DZSY21 suspensions (**Figure 4C**) and carbendazim wettable powder (**Figure 4D**) respectively could produce resistance phenotype characterized by the appearance of few small tan necrotic spots on the leaves.

The disease index of the maize leaves was calculated 4, 6, 8, 10, and 15 days after challenge with the pathogen, and was used to detect the resistant response of different groups. The results showed the disease index of the leaves was reduced in all inoculated plants pretreated with the DZSY21, lipopeptides and carbendazim wettable powder as compared with the negative control, and the resistant responses of DZSY21 and its lipopeptides were always better at different period (**Figure 4E**). At 8 days after challenge with the pathogen *B. maydis*, the disease index of DZSY21 and its lipopeptides were 15.60 and 19.30, respectively (**Figure 4E**), at the same time, the disease index of 50% carbendazim wettable powder was 32.80 (**Figure 4E**). Pre-treatment with DZSY21 and its lipopeptides also retarded disease development. At the 15 day time point, the disease index of DZSY21 and its lipopeptides were 33.50 and 36.42, respectively, compared to 40.78 for 50% carbendazim wettable powder (**Figure 4E**).

The disease reduction of the DZSY21 was evaluated in suppressing southern corn leaf blight development through the disease index assessment measure. The disease reduction was shown in all inoculated plants as compared with the negative control (**Table 2**). The disease reduction by strains DZSY21 and lipopeptides were 60.41% and 51.02% in 8 days after challenged-inoculation with the pathogen (**Table 2**). Pre-treatment with DZSY21, lipopeptides and carbendazim wettable powder also resulted in a slower progression of disease development. By day 15, the disease reduction of DZSY21, lipopeptides and carbendazim wettable were 42. 24, 37.24, and 31.03%, respectively (**Table 2**). Apparently the best disease reduction was achieved in the treatment with DZSY21, which showed significantly greater disease suppression than the chemical control. The results suggest that the inhibitory effect of lipopeptides is not significantly different than that of DZSY21, indicating that lipopeptides produced by DZSY21 could be the primary mechanism of disease suppression.

**Table 2:** The disease reduction of southern corn leaf blight after leaves treatment with strain DZSY21.

Treatment	Disease reduction (%)				
	4 days	6 days	8 days	10 days	15 days
DZSY21	65.87^a^	62.43^a^	60.41^a^	46.06^a^	42.24^a^
Lipopeptides	53.58^b^	54.24^b^	51.02^b^	42.02^b^	37.24^b^
50% carbendazim wettable powder	39.25^c^	29.66^c^	20.81^c^	27.47^c^	31.03^c^
Control	-	-	-	-	-

*Disease reduction was calculated at different days after challenge with *B. maydis*. “-” had no disease reduction as the control; different letters indicate significant differences between treatments according to Duncan’s multiple range test (α = 0.05). All experiments were performed at least three times.*

### Molecular Mass of DZSY21 Lipopeptides

**FIGURE 5:**
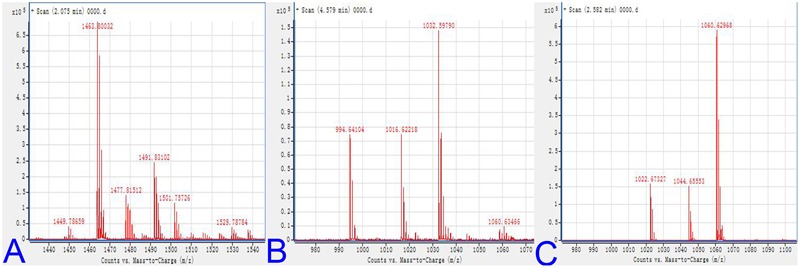
**MALDI-TOF mass spectra of lipopeptides: (A)** MALDI-TOF mass spectra of lipopeptides for the presence of antibiotic groups fengycin; **(B)** MALDI-TOF mass spectra of lipopeptides for the presence of antibiotic groups surfactin B; **(C)** MALDI-TOF mass spectra of lipopeptides for the presence of antibiotic groups surfactin A.

*Bacillus* species could produce a structurally diverse group of antimicrobial lipopeptides, including surfactin, iturin, and fengycin families (Wang et al., 2010b; Cai et al., 2013). The lipopeptides of DZSY21 was further characterized by MALDI-TOF-MS analysis for molecular mass and determination of lipopeptide groups. The DZSY21 lipopeptides contain members of the antifungal surfactin A, surfactin B, and fengycin families. The molecular mass of fengycin in the range m/z 1449.8–1491.8 was similar to previous published molecular mass (Pathak et al., 2012) (**Figure 5A**), mass spectra of fengycin including m/z 1449.8, 1463.8, 1477.8, and 1491.8 represented lipopeptide groups with different numbers of carbon atoms (m/z 14) in their fatty acid chains, and the compound at m/z 1449.8 represented a H adduct of fengycin (**Figure 5A**). The molecular mass of surfactin B was in the range of m/z 994.6–1032.6. These represented H, Na, and K adducts of surfactin B, respectively (**Figure 5B**), and were similar to previous published molecular masses (Mikkola et al., 2004). The other surfactin A was also confirmed by mass spectra of m/z 1022.6–1060.6, the mass spectra of m/z 1022.6–1060.6 represented H, Na, and K adducts of surfactin A, respectively (**Figure 5C**), the mass agrees with previous studies (Luo et al., 2015; Jiang et al., 2016). And the MALDI-TOF-MS characterization did not show any peaks corresponding to iturin lipopeptide.

### DZSY21 Mediates the Defense Response in Maize Plants

To determine the signaling pathways mediated by DZSY21, the expression of target plant genes known to function in the SA or JA/ET pathways were analyzed. Namely PR1 (an SA-responsive marker gene), defensin (PDF1.2) (JA/ET response marker gene), lipoxygenase (LOX) (a JA-responsive marker gene) and an ERF that could be expressed in the plant defense mechanism (Van Loon and Bakker, 2006; Pieterse et al., 2009) were used in this study. Maize leaves were harvested in the 12, 24, 36, 48, and 60 h separately after inoculation with DZSY21, and the changes in gene expression were analyzed by qRT-PCR.

**FIGURE 6:**
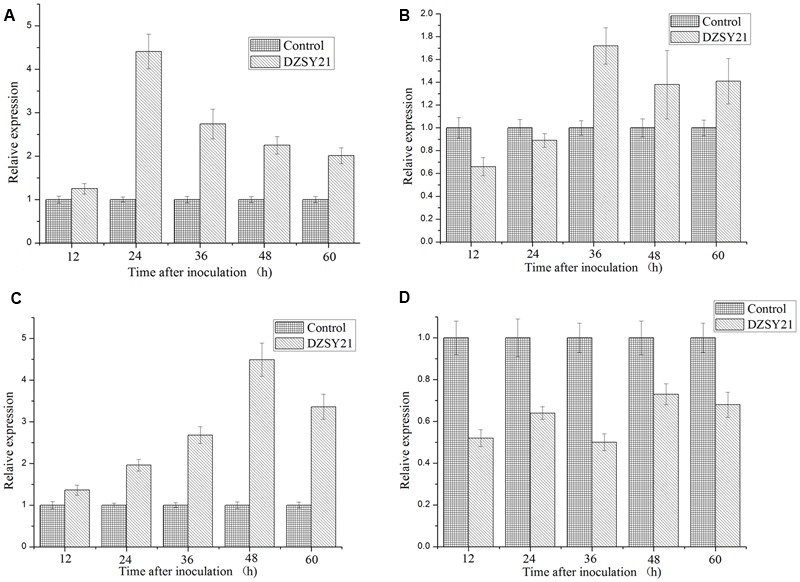
**Expression of the key genes involved in the salicylic acid (SA) or jasmonic acid (JA) and ethylene dependent defense signaling pathways in maize in different periods after inoculation. (A)** Expression of pathogenesis related protein (*PR1*); **(B)** Expression of plant defensing factor (*PDF 1.2*); **(C)** Expression of lipoxigenase (*LOX*); **(D)** Expression of epidermal repair factor (*ERF*). The graph shows expression levels of defense marker genes after normalization to the control gene actin. Data are expressed as the average of three replicates ± standard deviation.

We observed that the expression of *PR-1* was strongly induced in DZSY21- treated plants at levels 4.41- times in 24 h, compared to the control plants treated with water, then expression levels of *PR-1* were gradually reduced in 36, 48, and 60 h and the *PR-1* transcripts were induced to 2.74-, 2.25-, and 2.01-fold, respectively (**Figure 6A**). The *LOX* was gradually increased from 12 to 48 h after inoculation with DZSY21, and the expressions of *LOX* was strongly induced in DZSY21- treated plants with 4.49- times in 48 h, compared to the control plants (**Figure 6C**). The dynamic of *PDF1.2* expression in DZSY21 – treated plants were similar in the expressions of *LOX*, the *PDF1.2* transcripts were slightly induced to 1.72-, 1.38-, and 1.41-fold in 36, 48, and 60 h in DZSY21-treated plants, respectively (**Figure 6B**). Meanwhile, the expression of *ERF* in DZSY21-treated plants was lower than in the non-bacterized controls, and the higher expression of *ERF* was only 0.73 in 48 h after inoculation with DZSY21 (**Figure 6D**), indicating the expression was not enhanced in the presence of the DZSY21. These defense-related genes *PR1, LOX*, and *PDF1.2* were concurrently expressed in the leaves of DZSY21-treated plants, suggesting simultaneous activation of the salicylic acid (SA) -and the jasmonic acid (JA) -dependent signaling pathways by DZSY21. There was no evidence of any necrotic lesions in treated plants.

## Discussion

*Eucommia ulmoides* is a rare and precious plant, and it is not easily infected by plant diseases and insect pests, has a longlife time, and is used in Chinese traditional medicine. The endophytes isolated from *E. ulmoides* tissue could have useful functions, such as biocontrol and plant-growth-promoting activities and producing the same bioactive compounds like host plant (Chen et al., 2010; Liu et al., 2016). In this study, the biocontrol mechanisms of DZSY21 against southern corn leaf blight were evaluated. Our results could establish a framework for screening biocontrol strains and inform the design of appropriate protocols for using biocontrol strains.

The efficacy of endophytes as BCAs is dependent on many factors, including: host specificity, population dynamics, pattern of host colonization, and ability to move within host tissues (Backman et al., 1997). Endophytic bacteria need be grown robustly and a considerable population needs to be established in the internal plant tissues. DZSY21 isolated from *E. ulmoides* leaves were able to enter and colonize the internal leaves of maize, and were able to persist for 30 days. DZSY21 is also capable of colonizing other plants (i.e., lack of host specificity). It has been reported that colonization ability might be linked to certain factors, such as lipopolysaccharides, flagellas, and pili (Compant et al., 2010); further studies will be required to elucidate the mechanisms of colonization in DZSY21.

One of the most important modes of action for endophytic bacteria is antagonism mediated by different compounds with antifungal properties, especially the genus *Bacillus*. Lipopeptides, such as surfactin, bacillomycin, and fengycin, are major antimicrobial compounds secreted by *Bacillus* spp., and possess antifungal, antibacterial, immunosuppressive, antitumor, or other physiologically relevant bioactivities (Chen et al., 2008). In this study, the lipopeptides of DZSY21 belonging to *Bacillus* spp. showed antifungal activity and was highly effective in reducing disease index. The mycelium protoplasm of pathogen exposed to DZSY21 lipopeptides were deformed and contorted. The lipopeptides were characterized by MALDI-TOF analysis, and were found to contain members of the antifungal surfactin A, surfactin B, and fengycin families. Fengycin and surfactin are strong antifungal compounds secreted by *Bacillus* spp., which inhibit filamentous fungi by antagonizing sterols, phospholipids, and oleicacid in fungal membranes (Romero et al., 2007; Alvarez et al., 2012). Our results demonstrate that direct antifungal activity was the most dominant method of action of DZSY21 against southern corn leaf blight.

In addition to competition and direct antagonism, endophytic bacteria could control disease through indirect mechanisms. This includes ISR in the host plant, which involves a enhanced capacity to mobilize cellular defense responses before or upon pathogen challenge (Maryline et al., 2007; Verhagen et al., 2010) and induction of stress-related genes expression (Verhagen et al., 2004). ISR has been observed in some PGPB (Pieterse et al., 2001; Pahm et al., 2007; van Loon et al., 2009; Liu et al., 2010). Bacterial-mediated ISR involves elicitation of the ISR pathway, generation and translocation of the ISR signal, and ISR signal transduction leading to ISR-related gene expression and resistance (Pieterse et al., 2001; Liu et al., 2010).

In maize, there is little information regarding induction of the ISR pathway using endophytic microbes. Only a few studies have been reported showing inducing expression of defense-related genes in maize elicited by beneficial bacteria or exogenous JA (Van Loon and Bakker, 2006). Here, we identified some characteristic genes in maize that are similar to known or deduced functions involved in the SA and JA/ET pathways (Klosterman et al., 2011); these genes are suspected to play a role in generating signals for the activation of certain defense responses and protecting plants from damage associated with defense response. We found that defense-related genes *PR1* and *LOX* were highly expressed in the leaves of DZSY21-treated plants. However, the expression of *ERF* did not increase. Thus, *LOX* and *PR1* are likely responsible for conferring resistance against *B. maydis* infection in maize. Previously, *LOX* expression was shown to be stimulated by JA. The application of SA has been shown to trigger the expression of the PR1 gene. ISR is generally independent of the SA signaling pathway and is not associated with major alterations in the expression of defense-related genes, but is rather associated with the priming of defenses (Verhagen et al., 2004; van Loon et al., 2009). It has been well-established that there is cross-talk between the SA- and JA/ET-dependent signaling pathways (Koornneef and Pieterse, 2008), the SA and JA/ET signaling pathways interact antagonistically stimulating either one leads to the suppression of the other (Koornneef and Pieterse, 2008). However, the results in this study indicate that *LOX* and *PR1* are the defense-related genes responsible for conferring resistance on maize plants by both SA and JA pathways. Some studies have previously reported the induction of genes from both the SA and JA/ET pathways with endophytic microbes (Van Loon and Bakker, 2006), and Niu and associates found the *Bacillus cereus* AR156, a plant growth-promoting rhizobacterium, mediated ISR to *P. syringae* DC3000 in Arabidopsis through parallel activation of the SA- and JA/ET-signaling pathways, which leads to an additive effect on the level of induced disease resistance (Niu et al., 2011). Additionally, it has been reported that the *Bacillus* spp. elicit ISR in several plant species through enhanced peroxidise activity, increased production of chitinase isozymes and glucanase, and accumulation of SA (Kloepper et al., 2004). *B. thuringiensis* induced resistance to *R*. *solanacearum* in tomato plants through activation of the SA-dependent signaling pathway and suppression of the JA-dependent signaling pathway (Takahashi et al., 2014). Thus, some researchers have suggested that the specific ISR signal transduction pathway promoted by beneficial microbes is dependent on the strain, the host plant, and/or the pathogen.

In summary, we show that the suppression of southern corn leaf blight in maize by DZSY21 is a result of direct antagonism of antifungal lipopeptides produced by the DZSY21, as well as indirect inhibition through ISR. SA- and JA-mediated signal pathways are involved in the induction of ISR. Identifying the mechanisms of different biocontrol agents is important because helps to establish a theoretical basis for the design and appropriate use of biocontrol strains.

## Author Contributions

These studies were designed by TD, HJ. BS and XC carried out the major experimental analyses and prepared all figures and tables. SG, QW, and DH complete experiment of plant disease culture inoculation. TD analyzed the data and drafted the manuscript. SX contributed to revisions of the manuscript. HJ assisted in explaining the results and revised the final version of the manuscript. All authors have read and approved the final manuscript.

## Conflict of Interest Statement

The authors declare that the research was conducted in the absence of any commercial or financial relationships that could be construed as a potential conflict of interest.
